# Cocktail Protocol for Preparation of Platelet-Rich Fibrin Glue for Autologous Use

**DOI:** 10.21315/mjms2021.28.1.5

**Published:** 2021-02-24

**Authors:** Deepika Chenna, Shamee Shastry, Soumya Das

**Affiliations:** 1 Department of Immunohematology and Blood Transfusion, Kasturba Medical College of Manipal, Manipal Academy of Higher Education, Manipal, Karnataka, India; 2 Department of Transfusion Medicine, All India Institute of Medical Sciences, Nagpur, India

**Keywords:** platelets, biomaterials, conventional techniques, platelet-rich plasma, platelet-rich fibrin

## Abstract

**Background:**

Biomaterials containing platelets have been used to promote healing of ulcers and burns, as well as in implantology and maxillofacial and plastic surgery to achieve wound healing and tissue repair. Commercial devices to prepare autologous biomaterials involve diverse preparation methods that can have high production costs and low yields. Hence, we designed a protocol for preparation of large amounts of autologous platelet-rich fibrin (PRF) glue using conventional processing techniques for blood components.

**Methods:**

Autologous whole blood collected 72 h before surgery was processed to prepare platelet concentrates and cryoprecipitate. In a closed system, calcium was added to the cryoprecipitate to release autologous thrombin and generate a firm fibrin clot. The fibrin clot, platelets and calcium were then placed in a conical flask in which a PRF glue formed. The protocol was validated through determination of pre- and post-platelet counts and fibrinogen amounts in the product.

**Results:**

Platelets were recovered with 68% efficiency during the preparation. Essentially no platelets or fibrinogen were found in the supernatant of the PRF glue, suggesting that nearly all had been incorporated in a PRF glue having a relatively large (8 cm × 10 cm) size.

**Conclusion:**

The protocol described here is a cost-effective, simple and closed system that can be used to produce large-size PRF glue to promote repair of major surgical defects.

## Introduction

**Figure 1:**
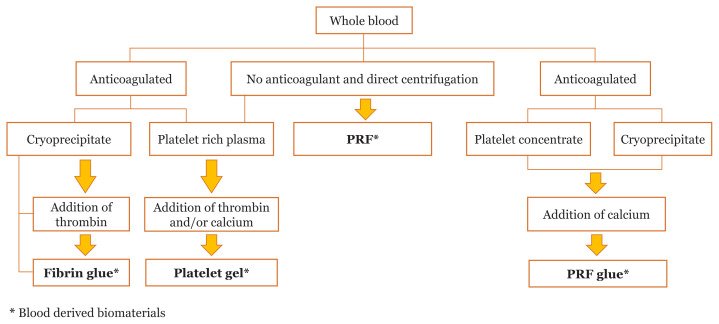
Preparation methods for various blood-derived biomaterials

Use of blood-derived biomaterials is gaining importance. Such materials can be prepared at the point of care, in transfusion medicine laboratories or at plasma fractionation centres. A variety of production methods and devices are used to produce biomaterials. These different techniques can affect the ultimate cellular and protein composition, which complicates the classification of these materials (Figure 1) (1, 2). Platelets are rich in growth factors that can accelerate tissue regeneration processes. Thus, platelets could accelerate healing of soft tissue and hard-tissue when incorporated in biomaterials for use in diverse applications including implantology, maxillo-facial surgery, treatment of osteoarthritis, sports medicine and treatment of ulcers or burns.

Several commercial systems are available for preparation of platelet gels at point-of-care centres (1–3). These systems are designed to allow processing and separation of 50 mL–100 mL of whole blood by centrifugation to yield platelet-rich plasma (PRP). These devices vary in terms of ease of use, number of processing steps, platelet recovery, and efficiency of platelet concentration and separation from red blood cells. Commercial devices to prepare autologous thrombin-enriched plasma have also been developed. However, the cost of production of these materials is substantially higher with respect to the amount of biomaterial that can be recovered using these techniques. Moreover, thrombin-enriched plasma does not provide sufficient coverage for large defects that can arise after evacuation of mandibular cysts or bony defects. Platelet-rich fibrin (PRF), a second-generation platelet concentrate, is simpler to prepare than PRP made using traditional methods and requires no biochemical processing of blood. Platelet gels obtained by mixing a platelet concentrate with thrombin or calcium salt solution results in conversion of the fibrinogen present in plasma into a fibrin gel simultaneously with platelet activation. However, the physiological content of fibrinogen and fibronectin in such gels is 2 g/L–3 g/L and 0.3 g/L–0.5 g/L, respectively, and these amounts would not provide sufficient tensile strength (4). Hence, enrichment of the platelet concentrate with fibrinogen (e.g. cryoprecipitate) prior to mixing with thrombin/calcium to produce the PRF glue would enhance both the tensile strength and the propensity of the gel to adhere to tissues. The fibrin framework could even protect growth factors from proteolysis, which would in turn preserve activity and prolong stimulation of effective regeneration (5).

Here we describe a protocol for preparation of autologous PRF glue that can be carried out using conventional blood component separation techniques.

## Methods

We established a procedure according to Good Manufacture Practice guidelines to prepare high-concentration, PRF from an autologous source for clinical applications in bone regeneration. The procedure was performed using samples from three patients who were referred by the Department of Oral and Maxillofacial Surgery after obtaining informed consent.

Indications for use of PRF glue correlated with physical examination and were confirmed by imaging studies. Contraindications for the procedure included platelet dysfunction syndrome, haemodynamic instability that could occur upon collection of large volume of blood, septicemia and local infection at the procedure site and consistent use of non-steroidal anti-inflammatory drugs (NSAIDs) within 48 h of the procedure were reviewed. The platelet count and the haemoglobin level of the patients were also analysed.

**Figure 2:**
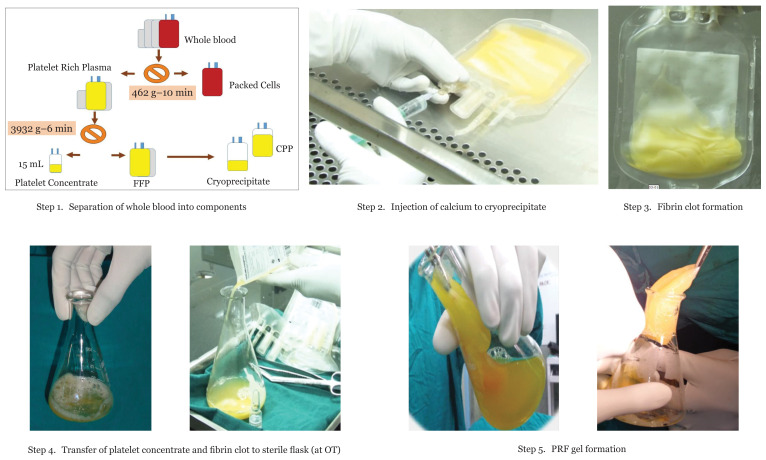
Steps of PRF glue preparation

Upon obtaining informed consent from the patients, autologous blood was collected 72 h before surgery. The volume of blood to be collected was dependent on the patient’s weight and did not exceed 9 mL/kg body weight. Blood was collected in a 350 mL quadruple bag and was centrifuged to separate red cells and plasma. The plasma was further centrifuged to obtain a platelet concentrate and platelet-poor plasma. Platelet concentrates were labelled and stored at 22 °C–24 °C and platelet-poor plasma was stored at −80 °C for preparation of cryoprecipitates. Frozen plasma is thawed at 4 °C after overnight hold and 20 mL–30 mL cryoprecipitate was prepared. On the day of surgery, calcium gluconate was added to the cryoprecipitate at a ratio of 1:5 in a biosafety cabinet and the mixture was then transferred to an incubator at 37 °C to allow formation of fibrin clots. The bag containing the fibrin clot along with the platelet concentrate and calcium gluconate was then taken to the operation theatre where the fibrin clot also acted as a source of autologous thrombin and platelet concentrate. Calcium gluconate was added to a conical flask. Within 60 sec–90 sec, a PRF glue of ~8 cm × 10 cm area formed and was immediately placed in the defect. The PRF glue acts as a source of growth factors and has an additional scaffolding function from the fibrin gel (Figure 2). The protocol was validated based on platelet and fibrinogen counts in the product before and after application. The platelet counts and fibrinogen concentration were also estimated at three steps during the preparation of the PRF glue to estimate the recovery of these components and to confirm its functionality. Platelet counts were estimated in the PRP, platelet concentrate and the supernatant of the platelet concentrate after activation. Fibrinogen concentration was estimated in fresh frozen plasma, the cryoprecipitate and in the supernatant after formation of PRF glue.

## Results

Quality control parameters were noted after every step of the procedure to validate the protocol (Table 1). The efficiency of platelet recovery was 68%. The recovery of the platelet count and fibrinogen content in the platelet concentrate and cryoprecipitate used for preparation of PRF glue was high (Table 1). Meanwhile, the estimated platelet counts and fibrinogen content in the supernatant that resulted after formation of the PRF glue were essentially zero, suggesting that nearly all the platelets and fibrinogen had been incorporated in the PRF glue.

### Major Advantages of the Protocol for Production of PRF Glue

The protocol for preparation of PRF glue involves a closed system, which reduces the potential for bacterial contamination. The risk from transfusion-transmitted infections is also minimal as the materials are from an autologous source. The use of endogenous thrombin minimises the risk that antibodies against bovine thrombin will develop. Overall, this protocol to prepare PRF glue is a simple and cost-effective procedure that requires no specialised equipment or training skills.

## Discussion

There is an urgent need for effective and economical products that can enhance tissue/wound healing as well as bone regeneration and repair. The therapeutic effect of such products is manifested through multiple growth factors that are released from alpha-granules found in activated human platelets. These growth factors include platelet-derived growth factor (PDGF), transforming growth factor (TGFb), fibroblast growth factor (FGF), epidermal growth factor (EGF), platelet-derived angiogenesis factor and β-thromboglobulin (6). Mitogenesis and angiogenesis, as well as macrophages can be activated by various growth factors to promote tissue repair and bone regeneration (6). The organisation of the fibrin matrix is higher in PRF and in turn can more efficiently direct stem cell migration and induction of healing pathways. Previous studies showed that cells can migrate from fibrin scaffolds and the PRF was also demonstrated to be a supportive matrix for bone morphogenetic protein (7).

The therapeutic osteogenic effect of local platelet administration likely depends on the number of growth factors delivered from the biomaterial, the length of time that growth factors are released, and the amount of fibrinogen that is available to protect the growth factors from proteolysis. PRP is a first generation platelet concentrate that has been used widely to accelerate healing of both soft and hard tissue. PRF preparation using the method described by Choukron et al. (8) involves centrifugation of whole blood collected in a 10 mL test tube without anticoagulant at 3,000 rpm for 10 min. Membranes composed of PRF can be formed by squeezing out fluids present in fibrin clots. This method and modified versions have been used in different clinical settings. For example, Mazor et al. (9) used multiple PRF membranes prepared from 72 mL whole blood collected in eight different test tubes as the only graft material used for sinus floor augmentation with simultaneous implant placement. Aroca et al. (10) used PRF prepared from 40 mL of whole blood in four test tubes each containing 10 mL for treatment of multiple adjacent gingival recessions. Other studies used whole blood collected in multiple test tubes to prepare PRF glue (11–13). All of these methods involved collection of whole blood in multiple test tubes to obtain the required amounts of PRF. However, the amount of fibrinogen in these preparations is substantially less than that achieved using the present protocol, which includes addition of cryoprecipitate that contains higher amounts of fibrinogen than in whole blood samples alone. The presence of fibrinogen both enhances tensile strength of the resulting biomaterial while protecting growth factors from degradation. Giannini et al. (6) used a distinctive technique to produce autologous PRF glue for treatment of maxillary and mandibular bone atrophy. In this method, a fibrin platelet gel was formed from dry platelets and cryoprecipitate (processed from plasma) collected by apheresis following addition of the thrombin-like material Botropase and calcium gluconate in a petri dish. However, the exogenous thrombin source used in this method could elicit antibodies against thrombin in the recipient (14). This adverse effect can be prevented by the use of the endogenous thrombin generation method described in this study.

## Limitation

Although we demonstrated that fibrinogen and platelets were incorporated into the fibrin clot, we did not estimate the amount of growth factors present in the glue.

## Conclusion

The protocol we developed is a cost-effective procedure that involves a closed system to produce biomaterials having sufficient size for repair of major defects. The simplicity and ease of preparation of PRF glue can be used even in resource-poor settings.

## Figures and Tables

**Table 1:** Quality control parameters

	PRP	Platelet concentrate	Supernatant
Mean platelet count	0.5 × 106/μL	1.2 × 106/μL	0
	**Fresh frozen plasma**	**Cryo**	**Supernatant**
Mean fibrinogen level	228 mg/dL	393 mg/dL	Undetectable
